# Agreement Marking May Facilitate Noun Class Learning in Children But Not Adults

**DOI:** 10.1162/OPMI.a.371

**Published:** 2026-07-17

**Authors:** Shira Tal, Kenny Smith, Jennifer Culbertson

**Affiliations:** Centre for Language Evolution, School of Philosophy, Psychology and Language Sciences, University of Edinburgh, Edinburgh, UK

**Keywords:** agreement, artificial language learning, language acquisition, learning biases

## Abstract

Agreement, defined as the systematic covariance between elements (e.g., between the 3rd person singular subject *she* and the -s verb ending in ‘she writes’), is present in many languages despite introducing redundant complexity. A prominent hypothesis suggests that the ubiquity of complex morphological features such as agreement might be attributable to a functional advantage it provides specifically to child learners (Lupyan & Dale, 2010). We tested this hypothesis through two artificial language learning experiments involving both children and adults. In both studies we compared noun class learning in artificial languages with and without agreement marking. While no overall advantage of agreement in noun classification was observed, our findings indicate that agreement may have a differential effect on children and adults. Specifically, agreement may facilitate the generalization of noun classification to novel nouns in children, but not in adults. These findings add to the growing body of evidence that highlights differences in the cues children and adults rely on in language acquisition. We discuss the implications of these results for theories of language acquisition and language change.

## INTRODUCTION

The world’s languages are replete with redundant elements – linguistic forms that add no additional information to the conveyed message (Dale & Lupyan, 2012; Leufkens, 2020; Mahowald et al., 2023; Pijpops & Zehentner, 2022; Wit & Gillette, 1999). In English, for example, verbs must match their subjects in person and number, as in ‘she writes’ rather than ‘she write’. The -*s* suffix on the verb is obligatory even though it is redundant: it repeats information (person and number) that is already conveyed by the subject. This is an instance of a widespread phenomenon across languages—*agreement*—a systematic formal mapping between related words.

The prevalence of agreement, and linguistic redundancy more generally, has been a longstanding puzzle; it seems to make languages more complicated. There is evidence that adding more components to a linguistic system can make it harder to learn and use (Dahl, 2004; Hawkins, 2004; Kusters, 2008; Miestamo, 2006, 2008). Furthermore, because the increase in complexity does not come with added information, it seems to go against a robust principle of language design: efficiency (Gibson et al., 2019; Levshina, 2021; Mahowald et al., 2023). There is a large body of evidence suggesting that languages are shaped both by a pressure to reduce complexity in general, and by a pressure for efficiency, i.e., to maintain complexity only when it is communicatively necessary (Dryer, 2013; Gibson et al., 2019; Kirby et al., 2015; Levshina & Moran, 2021; Mahowald et al., 2022; Zaslavsky et al., 2018). For example, in both natural language corpus data and in experiments using artificial language tasks, speakers tend to minimize redundancy by omitting or reducing linguistic material that adds no additional information (Caballero & Kapatsinski, 2015; Dahl, 2004; Mahowald et al., 2013; Pate & Goldwater, 2015; Smith & Culbertson, 2025; Tily & Piantadosi, 2009). Why, then, is agreement such a common feature of language despite apparently increasing complexity and running counter to speakers’ tendency toward efficiency? In this paper, we explore the hypothesis that agreement, as an instance of linguistic redundancy, might actually benefit learning, particularly among child learners. Before describing the current proposal in detail, we review various theories posited in the literature regarding the functional benefits of redundancy.

### Possible Advantages for Redundancy in Language

The possible functionality of redundancy in languages is a long-debated topic in the language sciences (Dahl, 2004; Leufkens, 2020; Wit & Gillette, 1999). One proposal is that redundancy makes linguistic signals more *robust*: in the presence of noise, having more than one cue for a certain meaning increases the chances of succesful communication (Gibson et al., 2019; Monaghan, 2017). For instance, in Choguita Rarámuri (an Uto-Aztecan language spoken in northern Mexico), some meanings are cued by two consecutive suffixes, the second of which is optional. Caballero and Kapatsinski (2015) investigated the impact of these redundant morphemes on word recognition among speakers of this language. They found that having a redundant morpheme aided recognition for ambiguous words, but made it *worse* for unambiguous words. That is, Choguita Rarámuri speakers expect a redundant morpheme only when understandability is otherwise compromised. This is in line with principles of efficient communication: speakers tend to increase redundancy when the conveyed meaning is less predictable in context (Kurumada & Jaeger, 2015; Levshina & Moran, 2021; Mahowald et al., 2013).

In some cases, linguistic redundancy can aid real-time processing by making upcoming linguistic information more predictable. For example, in German, nouns follow determiners that agree with them in gender (see (1)). Since the determiner precedes the noun, it reduces the set of possible following nouns by cueing which gender class the noun belongs to (Dye et al., 2017). Indeed, native speakers make use of this information, by ruling out irrelevant nouns—ones that have a different gender—before they process the noun itself (for evidence from other languages see Dahan et al., 2000; Grosjean et al., 1994; Lew-Williams & Fernald, 2007).

(1) German (Dye et al., 2017)
    Gestern besuchte ich den   Arzt
    Yesterday visited I the.MASC doctor

Finally, another proposed advantage of redundancy is its potential to benefit *learning*, by providing multiple cues for the same piece of information (Audring, 2014; Bates & MacWhinney, 1989; Lupyan & Dale, 2010; Monaghan, 2017; Tal & Arnon, 2022). A growing body of work demonstrates that language learners benefit from redundant multi-modal cues (Flom & Bahrick, 2010; Lew-Williams et al., 2019; Monaghan, 2017; Murgiano et al., 2021). For example, synchronous touch cues applied to specific body parts help infants segment words from continuous speech (Seidl et al., 2015). However, the facilitating effect of redundant multi-modal cues does not necessarily extend to redundant morphological cues, such as agreement. While redundant morphemes may provide additional cues that can be beneficial, they also introduce a cost: these morphemes themselves, and the agreement rules governing the mapping between agreeing elements, must be learned (Bates & MacWhinney, 1989; Leufkens, 2020). For this reason, redundant morphology is typically argued to render languages more complicated (Bentz et al., 2016; Dahl, 2004; Lupyan & Dale, 2010). Despite this added cost, recent findings support the hypothesis that morphological redundancy can benefit learning, and more specifically, child learners (Tal & Arnon, 2022). Using an artificial language learning paradigm, Tal and Arnon (2022) compared the learnability of two versions of an otherwise identical language: one where word order alone provides a cue to thematic role assignment (i.e., understanding who did what to whom), and one where there is an additional, redundant, case-marking cue. Participants who were exposed to the language with the redundant cue showed better comprehension of thematic assignment, despite having to learn and reproduce an additional morpheme. While no difference was found between children and adults, post-hoc analyses for each age group separately revealed that children showed better learning in the redundant language version whereas adults showed no difference between the language versions^1^. Importantly, however, adults were close to ceiling in both conditions, and therefore it is unclear whether they in fact differed from children. Crucially, a recent hypothesis predicts that such differences between children and adults should be found.

### Child Learners vs. Adult Learners

Children and adults are known to learn languages differently, likely for a number of reasons. They receive different amounts and types of linguistic input (Soderstrom, 2007; Uther et al., 2007), have different experience with languages (Arnon & Christiansen, 2017; Siegelman & Arnon, 2015), and different experience with the world. Moreover, children and adults generally differ in some aspects of their cognitive skills and neurological architecture (Kuhl, 2000; Neville & Bavelier, 2001). Consequently, they often show different learning strategies and biases when learning language (Culbertson et al., 2019; Hudson Kam & Newport, 2005, 2009; Raviv & Arnon, 2018; Samara et al., 2017; Schwab et al., 2018). For example, when learning a novel artificial language with a grammatical gender, or noun class-like system, Culbertson et al. (2019) found that adult learners more readily rely on extralinguistic cues, like the meaning of a word, but child learners are more sensitive to intralinguistic cues, that is, cues that are embedded within the linguistic signal itself (Culbertson et al., 2019; see also Gagliardi & Lidz, 2014; Karmiloff-Smith, 1981). These cues are exactly the kind that are found in patterns of agreement in general. Such differences may thus impact what adults and children learn about morphology. Indeed, there is evidence that adult learners struggle with acquiring various types of complex morphology, even when similar features exist in their native language (Clahsen et al., 2010; DeKeyser, 2005; Ellis, 2022). For example, adult learners of Turkish have difficulty acquiring case-marking morphology, even though their native language has rich morphology, including case marking (Papadopoulou et al., 2011).

In an influential paper, Lupyan and Dale (2010) argue that these learning differences progressively shape the relative complexity of languages: languages used by many adult learners lose morphological complexity over time (see also Bentz et al., 2015; Bentz & Winter, 2013; Dale & Lupyan, 2012; Trudgill, 2009; though see Shcherbakova et al., 2023 for counter evidence for certain morphological features, not redundant morphology). Lupyan and Dale (2010) provide correlational evidence for this hypothesis by showing that languages used by a higher proportion of adult learners are less likely to possess noun or verb agreement. A corollary hypothesis is that the reason why redundant morphology is attested in languages to begin with is its potential *advantage* for child learners (Lupyan & Dale, 2010). That is, while adult learners struggle with redundant morphology, child learners are predicted to benefit from it. The findings of Tal and Arnon (2022) support the hypothesis that morphological redundancy can benefit child learners. However, because adults in Tal and Arnon (2022) were at ceiling in their task, it is still unclear whether children and adults are in fact *differentially* influenced by morphological redundancy. If the prevalence of agreement markers is linked to the advantage it holds for child learners but not adult learners, then child learners should benefit from it, but adults should not.

Here, following Tal and Arnon (2022), we use an artificial language learning paradigm to compare the impact of redundant morphology on both types of learners. However, rather than focusing on case marking, we compare learning of a noun class system in the presence or absence of a redundant agreement cue. While agreement might impact learning of multiple features of a language (see discussion in Leufkens, 2020), it has been specifically hypothesized to facilitate learning of noun class systems in particular (Audring, 2008, 2014). We further examine whether the presence of a redundant agreement marker is more beneficial when noun classification becomes more challenging. In particular, it has been argued that redundant agreement marking may be more necessary in noun class systems that are not purely semantic (Audring, 2008). To test this, in Study 1 we compared classification of nouns with and without a semantic cue to class. In Study 2 we examined another dimension of challenge: we compared nouns that participants encountered during training with novel nouns that required generalisation.

In Study 1 we tested adults, who were tasked with learning a relatively complex noun class system, with no agreement, or one of two types of agreement system. To preview, we found that having agreement present in the language did not make the noun classes easier to learn (though unsurprisingly, the agreement patterns themselves were easier to learn when they involved repetition of form across agreeing elements). This null result is in line with the hypothesis that agreement, as an instance of redundant morphology, does not benefit adult learners. In Study 2 we tested both children and adults on a simpler language. While we again found no evidence that agreement benefits noun class learning per se, we did find some evidence for learning differences between children and adults in how they were impacted by the agreement.

## STUDY 1: ADULT LEARNERS

In Study 1, we investigate whether adult learners can benefit from a redundant agreement marker when learning noun classes in a novel language. Specifically, we compare learning from two types of agreement that are attested in the world’s languages and indeed fairly common: alliterative and non-alliterative. In alliterative agreement, the agreement is expressed by repetition of the same form. In contrast, in non-alliterative agreement (sometimes referred to as *arbitrary* or *opaque* agreement, Corbett, 2006) it is expressed by different forms. Swahili, for example, shows both types of agreement for different gender classes (Examples 2(a) and 2(b) respectively).

(2) Swahili (Bantu) (Corbett, 2006; Güldemann & Fiedler, 2021)
  - ki-ti  ki-le  ki-moja ki-me-anguka
      7-chair 7-D.DEM 7-one 7-PERF-fall
    ‘that one chair has fallen’
  - m-shale  u-lianguka
    3/4-SG-nail 3-fell
    ‘a nail fell’

Importantly, although both types of agreement systems are redundant, they pose different predictions with respect to learning. In particular, while both provide additional cues for the same information, these cues seem to have different costs. Alliterative agreement systems should be less costly for learners since, first, they require fewer forms to learn (Bates & MacWhinney, 1989) and, second, they are repetitive—a characteristic that typically facilitates language learning (Horst et al., 2011; Nencheva et al., 2024; Ota & Skarabela, 2016). Taken together, if agreement can benefit adult learners, then learning should be improved by both types of agreement, but more so by alliterative agreement.

### Methods

#### Participants.

Participants were recruited through Prolific. 150 adults, self-reported native speakers of English, took part in the study (50 in the no-agreement condition, 50 in the alliterative condition, 50 in the non-alliterative condition). In order to be included in the analysis, participants had to achieve a mean overall accuracy level of at least 80% in the attention tests (see below). All 150 participants met this threshold.

#### Materials.

Across all conditions, participants were exposed to an artificial language comprising 18 nouns, referring to animals, plants, or objects. Nouns were divided into two noun classes, indicated by two distinct noun suffixes. Within each class, six nouns had an additional semantic cue; they referred to either animals or plants, depending on the class. The remaining three nouns in each noun class referred to inanimate objects, and thus provided no semantic cue. Therefore, for a subset of nouns in each class, there were two sources of evidence for noun classification.

For each participant, we randomly selected 18 referents (6 animal, 6 plant, 6 objects; 3 per noun class) from a larger set of 42 possible referents to use during the exposure phase (see Appendix A for the full list of referents). The remaining 24 referents (12 animals, 12 plants) were used as novel nouns in testing (see below for details). A noun label for each referent was chosen randomly from a set of 42 monosyllabic nonsense words^2^ (see Appendix B for a list of all possible words in the language).

Participants were presented with images showing a single referent, described using a bare noun, and images showing a pair of the same referents, described using a modifying numeral plus noun, where the numeral always preceded the noun. Singular nouns were identical for all conditions, i.e., they always featured the noun class suffix. In the agreement conditions, numerals additionally had suffixes that agreed with the noun suffixes; in the alliterative agreement condition the two agreeing suffixes were identical to the two noun suffixes, whereas in the non-alliterative agreement condition, the two agreeing suffixes were different from the two noun suffixes (see Table 1 for example stimuli in all conditions). The language was displayed both orthographically and auditorily to participants.

**Table 1.** Example input structure for the three experimental conditions in Study 1.

	**No agreement**	**Alliterative agreement**	**Non-alliterative agreement**	
Class 1	glurn cridpa	glurnpa cridpa	glurnki cridpa	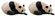
Class 2	glurn tojmu	glurnmu tojmu	glurnze tojmu	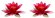

The numeral label was randomly selected for each participant from the set {*gleaf*, *glurn*}. There were 4 possible labels for suffixes: *ki*, *pa*, *mu*, *ze*. In the no-agreement condition, two suffixes were randomly selected for each participant to indicate the two noun classes on the nouns themselves (e.g., *glurn cridpa* vs. *glurn tojmu*). In the alliterative agreement condition, two suffixes were randomly selected for each participant, and these were used both on the nouns themselves and the numeral (e.g., *glurnpa cridpa* vs. *glurnmu tojmu*). In the non-alliterative agreement condition, all four suffixes were used and their assignment to classes and to noun or numeral was randomized for each participant (e.g., *glurnki cridpa* vs. *glurnze tojmu*).

#### Procedure.

The experiment was conducted online via code running in the web browser (written in JavaScript and using JsPsych; de Leeuw, 2015). Participants were told they were going to learn part of a new language. The experiment consisted of three training phases and three tests. The training phases included interspersed attention tests, that served two purposes: monitoring participants’ engagement and familiarizing them with the tests they would encounter after training. Some attention tests appeared only in the agreement conditions, since the task involved the agreement pattern (as detailed below). Participants received feedback on the attention tests.

**Figure 1.**
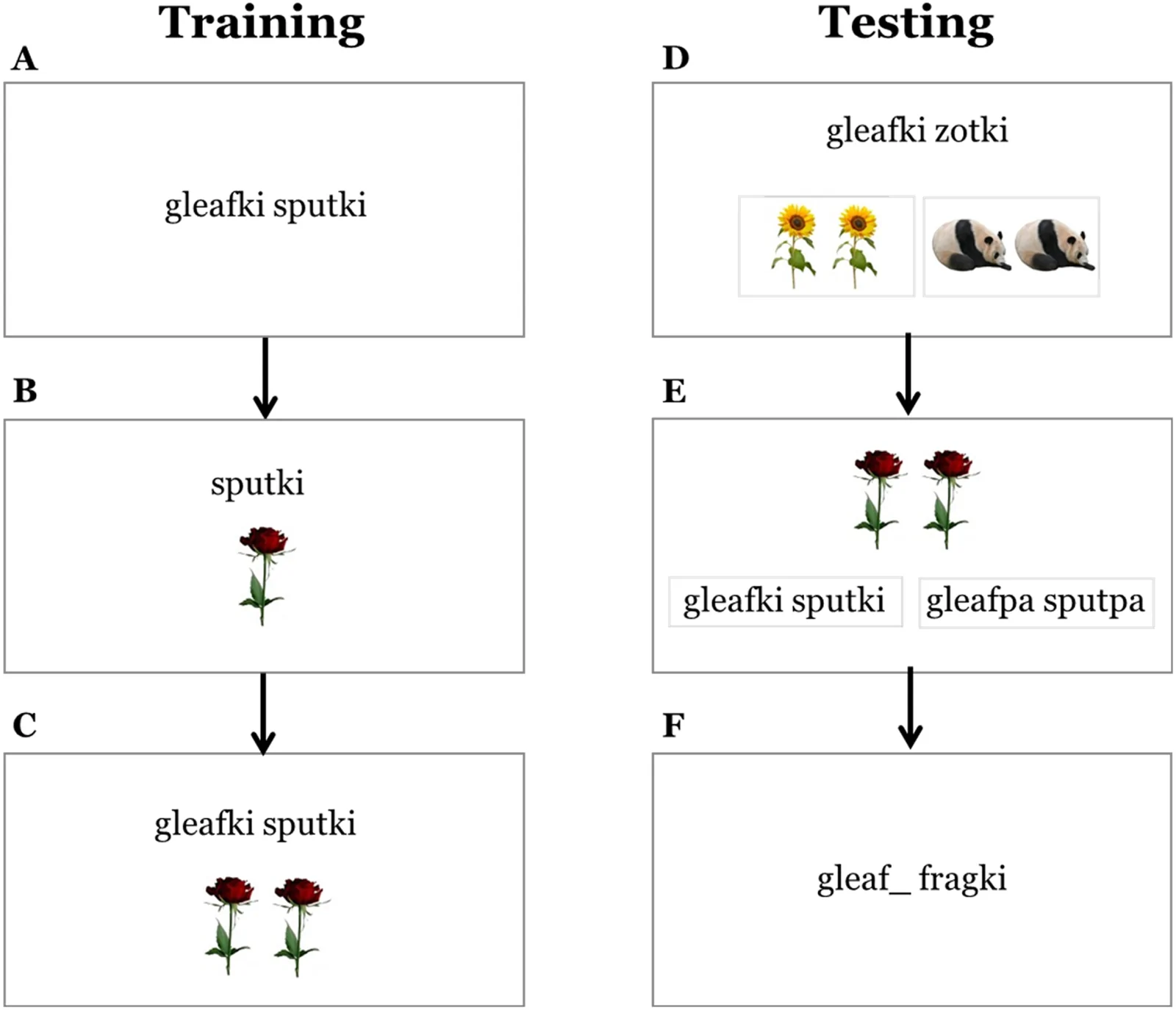
Experimental procedure for Study 1, illustrating the alliterative-agreement condition. The experiment began with three training phases (A, B, C). Following training there were three testing phases: *picture matching test*, *noun classification test*, and *agreement test* (D, E, F respectively), with the final phase (F) exclusive to the agreement conditions.

In the first training phase, participants were exposed to the nouns, in audio and text, without their corresponding referents (see Figure 1A). This was done following previous studies showing that exposure to phonological cues before semantic cues can boost learning (Culbertson et al., 2019). Nouns appeared either in singular (i.e., bare) or modified by a numeral. There were 36 trials in this phase (each noun appeared once in the singular and once with the numeral). Attention tests were randomly interspersed among these 36 trials: three randomly selected singular nouns trials were followed by an attention test, where participants were shown the previously presented noun with a missing suffix, along with two suffix options, one for each noun class in their input, and had to choose the correct suffix. Participants in the agreement conditions had 4 additional attention tests following four randomly chosen numeral plus noun trials. In these tests, they were presented with the previously presented phrase, but with a missing suffix on the numeral. They were given two possible numeral-suffix options from their input and had to select the correct one.

In the next two training phases, the same nouns appeared again, but now along with their referents. In the second training phase, nouns occurred only in the singular. There were 36 trials in this phase (each singular noun appeared twice). On each trial, a singular noun was presented alongside its corresponding referent—a single object (e.g., *sputki* alongside a picture of a rose, see Figure 1B). Seven randomly selected trials were followed by attention tests. In these tests, participants were shown the previously presented referent and its corresponding noun with a missing suffix. The two possible noun-suffix options were presented, and participants had to select the correct one.

In the third and final training phase, participants were exposed again to the same nouns alongside their referents, but this time nouns only appeared with numerals. On each trial, a numeral-modified construction was presented alongside its corresponding referent—an object appearing twice (e.g., *gleafki sputki* alongside a picture of two roses, see Figure 1C). There were 36 trials in this phase (each noun appeared twice).

The first testing phase involved a *picture-matching test* that assessed how well participants generalized the semantic cue—applicable to the majority of nouns in each noun class—to novel nouns sharing the same semantic cue. On each trial, participants were presented with a novel noun stem combined with one of the noun class suffixes, accompanied by the modifying numeral. Participants were then shown two novel images: one depicting two animals and the other depicting two plants. Their task was to select the image that best matched the presented noun description (see Figure 1D). This phase included 12 trials, one per novel noun.

The following phase, the *noun classification test*, tested noun class learning with nouns seen during exposure. On each trial, participants were presented with one of the objects they had learned, modified by a numeral (e.g., two roses). They were given two descriptive options to choose from. Both descriptions included the correct noun stem, but they differed with respect to the suffixes: one had the correct suffix(es), and one had the suffix(es) of the wrong class (e.g., *gleafki sputki* vs. *gleafpa sputpa*, see Figure 1E). This phase included 18 trials (one per noun)^3^.

Finally, to test learning of the agreement patterns themselves, participants in the agreement conditions went through an additional testing phase, the *agreement test*. In each trial they were presented with suffixed nouns and were asked to provide the corresponding suffixed numeral (e.g., *gleaf_fragki*, see Figure 1F). In order to test the acquisition of the agreement pattern in the absence of other cues, all noun stems were new, and no referents were presented on the screen. There were 12 such trials, one per novel noun.

##### Analytic Approach.

All analyses reported here used Bayesian mixed-effects logistic regression models with a Bernoulli likelihood, implemented with the brms package (Bürkner, 2017) in R software (R Core Team, 2020). Models used brms default priors: flat priors for fixed effects, weakly informative Student-t priors (df = 3, mean = 0, scale = 2.5) for the intercept and random effect standard deviations, and LKJ(1) priors for random effect correlations. For each model we report posterior means and 95% credible intervals (CrI) on the log-odds scale. To aid interpretation, we additionally report predicted differences in accuracy, expressed in percentage points (pp), and the posterior probability of direction (pd): the proportion of the posterior distribution above zero for positive effects, or below zero for negative effects, reflecting how consistently the data point toward one direction.

### Results

All 150 participants scored above the 80% threshold in the attention tests (mean accuracy 99% in the no agreement condition; 98% in the alliterative-agreement condition; 97% in the non-alliterative-agreement condition).

**Figure 2.**
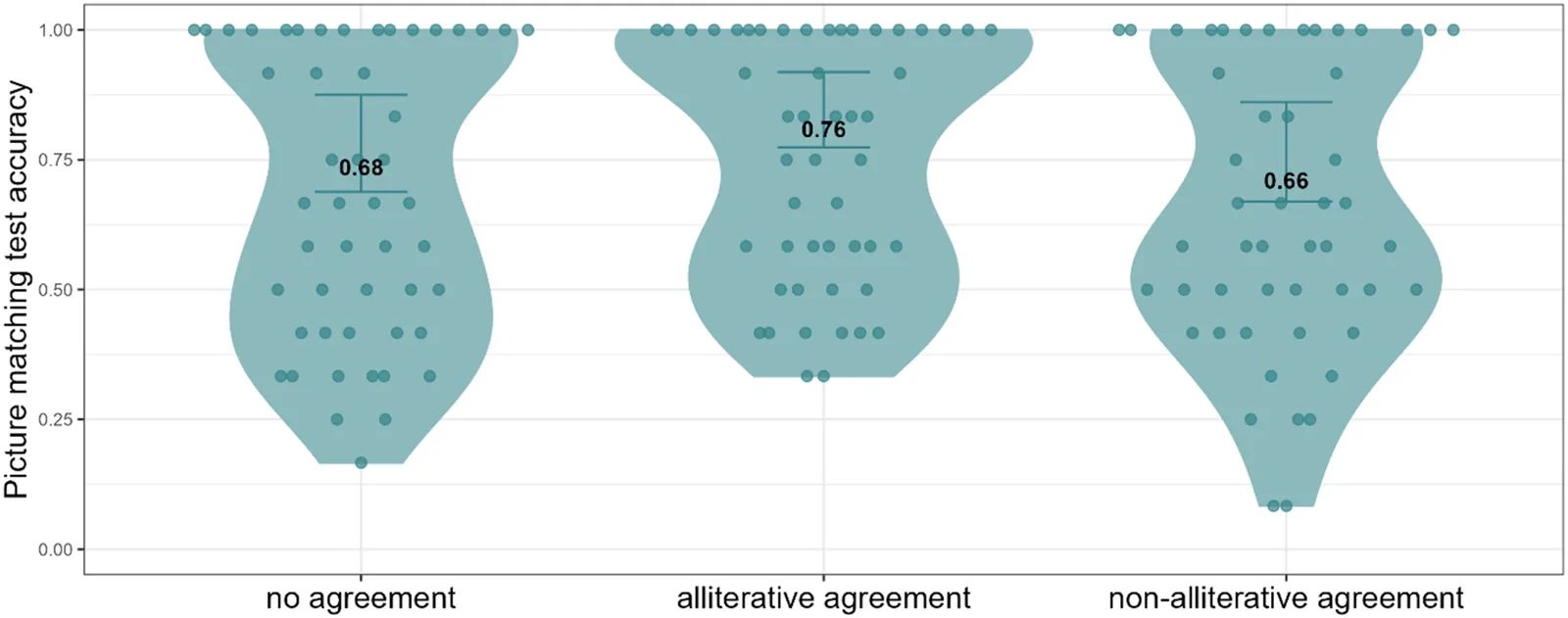
Accuracy scores in the picture matching test by language condition. Error bars indicate the model’s predicted 95% credible intervals; individual points indicate by-participant means; numbers represent group means.

Figure 2 shows participants’ scores in the *picture matching test* (Figure 1D). We modelled accuracy, with fixed effects for condition (treatment coded, with no-agreement condition as the baseline level) and trial number (centered), random by-participant slopes for trial number and random intercepts for participants. The fitted model’s posteriors are summarized in Table 2. For the alliterative agreement condition, the posterior favoured a positive direction relative to no-agreement (6.96 pp; 95% CrI: [−4.42, 18.6] pp; log−odds: *β* = 0.49, 95% CrI = [−0.32, 1.26], pd = 0.89). For the non-alliterative agreement condition, the posterior showed only a weak tendency toward negative values (−1.53 pp; 95% CrI: [−14.2, 11.08] pp; log-odds: *β* = −0.09, 95% CrI = [−0.84, 0.65], pd = 0.596), providing no evidence of a systematic effect in either direction. Taken together, while the alliterative condition shows a tendency in the expected direction, the pattern does not extend to the non-alliterative condition, and the overall evidence for agreement facilitating generalisation of learned noun classes via semantic cues remains uncertain. Nevertheless, participants in all three conditions were robustly above chance performance (78.7 pp, 95% CrI: [68.8, 87.5] pp; log-odds: *β* = 1.331, 95% CrI = [0.79, 1.95], pd = 1) indicating that the semantic cue to noun class was learned and could be generalised to novel nouns.

**Table 2.** Posterior means and 95% CrIs for the picture matching test in Study 1.

	**Estimate**	**Est’d Error**	**Lower 95% CrI**	**Upper 95% CrI**
(Intercept)	1.331	0.296	0.793	1.947
Condition = alliterative agreement	0.488	0.397	−0.315	1.256
Condition = non-alliterative agreement	−0.092	0.382	−0.841	0.646
Trial number	0.053	0.03	−0.004	0.114

**Figure 3.**
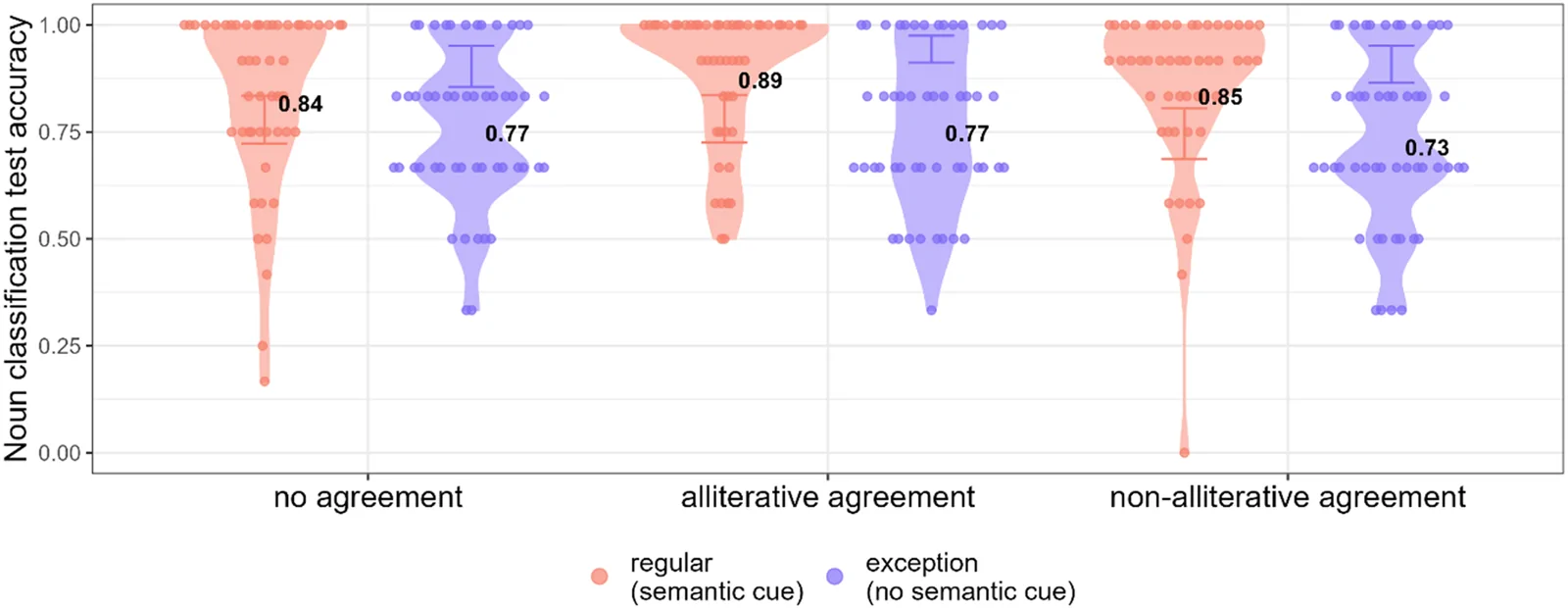
Accuracy scores in the noun classification test by language condition and noun type. Error bars indicate the model’s predicted 95% credible intervals; individual points indicate by-participant means; numbers represent group means.

We next analysed accuracy in the *noun classification test* (Figure 1E). Recall that in this test, participants saw a picture and had to choose the phrase with the correct suffix(es). Figure 3 shows participants’ accuracy scores across conditions and noun types (nouns with a semantic cue, e.g., rose, vs. nouns without a semantic cue, e.g., pan). The model included fixed effects for condition (treatment coded, with no-agreement condition as the baseline level), noun type (i.e., whether the noun had a semantic cue; treatment coded, with no semantic cue as the baseline level), the interaction of these two fixed effects, and trial number (centered). The model further included random intercepts for participants and by-participant slopes for noun type and trial number (see Table 3 for full model). We observed a positive effect of noun type in the baseline no-agreement condition, with semantically cued nouns classified more accurately than exception nouns (12.9 pp, 95% CrI: [6.6, 19.2] pp; log-odds: *β* = 1.07, 95% CrI = [0.51, 1.68]). To interpret this effect across all conditions, we calculated the average marginal effect of semantic cues, marginalizing over condition levels. This yielded a robust positive effect (15.0 pp, 95% CrI: [10.6, 19.9] pp; log-odds: *β* = 1.34, 95% CrI = [0.96, 1.73], pd = 1), with the credible interval entirely above zero. Overall, this indicates that when semantic cues provide an additional source of evidence, they can benefit learning. In contrast, neither agreement condition differed meaningfully from the no-agreement baseline. For the alliterative-agreement condition, the posterior was approximately evenly split around zero (0.21 pp, 95% CrI: [−7.5, 7.7] pp; log-odds: *β* = 0.01, 95% CrI = [−0.43, 0.46], pd = 52.8). For the non-alliterative agreement condition, the posterior showed a modest tendency toward negative values (−3.3 pp, 95% CrI: [−11.29, 4.37] pp; log-odds: *β* = −0.19, 95% CrI = [−0.64, 0.25], pd = 79.8). Taken together, while semantic cues reliably benefited noun class learning, there was no comparable evidence for a benefit from *morphological* agreement cues, regardless of agreement type.

**Table 3.** Posterior means and 95% CrIs for noun classification test in Study 1.

	**Estimate**	**Est’d Error**	**Lower 95% CrI**	**Upper 95% CrI**
(Intercept)	1.28	0.167	0.959	1.617
Condition = alliterative agreement	0.013	0.229	−0.435	0.457
Condition = non-alliterative agreement	−0.185	0.224	−0.638	0.248
Noun type = semantic cue	1.075	0.304	0.505	1.676
Trial number	0.011	0.013	−0.013	0.036
Condition = alliterative agreement * Noun type = semantic cue	0.585	0.41	−0.205	1.412
Condition = non-alliterative agreement * Noun type = semantic cue	0.237	0.4	−0.544	1.029

**Figure 4.**
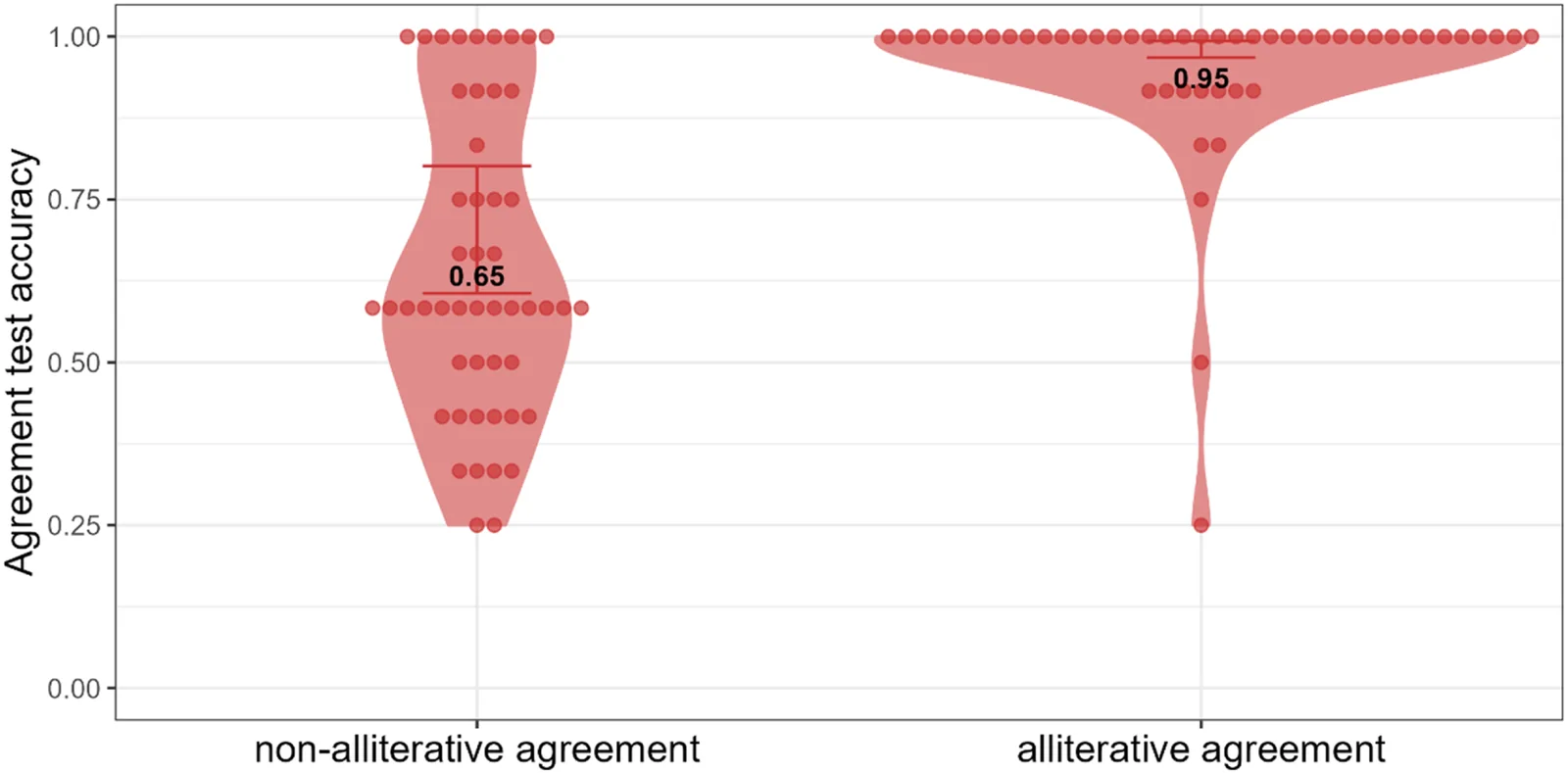
Accuracy scores in the agreement test by language condition. Error bars indicate the model’s predicted 95% credible intervals; individual points indicate by-participant means; numbers represent group means.

Finally, we analysed the agreement test, which tested how well participants in the agreement conditions learned the agreement patterns themselves. Accuracy in this test across both agreement conditions is presented in Figure 4. This model included only the two agreement conditions (treatment coded, with the no-alliterative agreement condition as the baseline level) and trial number (centered). The model also included by-participant intercepts and by-participant slopes for trial number (see Table 4 for the full model).

**Table 4.** Posterior means and 95% CrIs for agreement test in Study 1.

	**Estimate**	**Est’d Error**	**Lower 95% CrI**	**Upper 95% CrI**
(Intercept)	0.92	0.25	0.431	1.396
Condition = alliterative agreement	3.215	0.457	2.38	4.202
Trial number	0.048	0.032	−0.013	0.115

The model’s intercept reflects accuracy in the non-alliterative agreement condition. With its 95% credible interval entirely above zero (71.23 pp, 95% CrI: [60.61, 80.15] pp; log-odds: *β* = 0.92, 95% CrI = [0.43, 1.40], pd = 1), the model indicates that participants performed above chance in this condition. Accuracy was however substantially higher in the alliterative-agreement condition compared to the non-alliterative agreement condition (27.1 pp, 95% CrI: [18.3, 37.6] pp; log-odds: *β* = 3.21, 95% CrI = [2.38, 4.20], pd = 1). This suggests that alliterative cues were considerably more readily learned than non-alliterative cues.

### Discussion

The results of Study 1 show that while alliterative agreement showed a modest tendency to benefit the generalisation of semantic cues to novel nouns in the picture matching task, the presence of redundant agreement marking did not benefit learning of noun classification, regardless of whether additional semantic cues were available. However, in terms of learning the agreement markers themselves, the alliterative type, featuring repeating forms, was easier to learn than the non-alliterative type. This result is consistent with the relatively lower learning cost associated with repetitive forms (Nencheva et al., 2024; Ota & Skarabela, 2016).

Taken together, these findings are in line with previous work showing that adults struggle with redundant morphology (Clahsen et al., 2010; DeKeyser, 2005; Ellis, 2022). In the non-alliterative condition in particular, the mapping between noun and numeral suffixes was clearly very difficult for participants, with mean accuracy on a 2-alternative agreement test of 65%. This, together with the fact that noun class performance was the same across agreement conditions, suggests that adults may have largely been using the semantic cues, rather than suffixes. When no semantic cue was present, i.e., in object trials, adults may have been relying to some degree on memory for individual object meanings to determine class. Importantly, while redundant morphology is difficult for adult learners, and may not be utilised as readily as meaning (Culbertson et al., 2019), as mentioned above, it could benefit *child* learners (Lupyan & Dale, 2010; Tal & Arnon, 2022). If so, then child learners should benefit from the presence of agreement markers even when adult learners do not. To test this, in Study 2 we ran a simplified version of Study 1 on both children and adults.

## STUDY 2: COMPARING CHILD AND ADULT LEARNERS

Study 2 aimed to investigate whether child learners, unlike adult learners, benefit from a redundant agreement marker when learning noun classes in a novel language. This study used a similar artificial language learning experiment, but with a simpler language and child-friendly referents (see details below). Moreover, following the results from Study 1, which showed that alliterative agreement markers were easier to learn than non-alliterative ones, we simplified the task for children by only comparing the alliterative-agreement condition (hereafter referred to as the agreement condition) and the no-agreement condition. As in Study 1, we examined whether the presence of an agreement marker could facilitate noun class learning, and whether this benefit is modulated by the sources of evidence available for noun classification. Specifically, we tested both nouns that participants encountered during training and novel nouns that required generalisation.

### Methods

#### Participants.

56 children (age range: 5;0–7;0, mean age: 5;11) and 100 adults took part in the study. Child participants were recruited through social media and conducted the study online via Zoom (see details below). Parental consent was obtained for all children. Adult participants were recruited through Prolific. All participants were native English speakers, and none of them had known language or learning disabilities. In each age group participants were randomly assigned to one of the two language conditions: the agreement condition (28 child participants, 50 adult participants) and the no-agreement language (28 child participants, 50 adult participants).

#### Materials.

The artificial language (a modified version of the language used in Culbertson et al., 2019) consisted of 12 nouns, referring to 6 aliens and 6 planets. The nouns were divided into two noun classes, as in Study 1, indicated by distinct noun suffixes (e.g., *po* vs. *fei*). In addition, a semantic cue was present for all nouns (aliens vs. planets), and internal phonological features of noun stems also cued class membership (e.g., *mibi* vs. *gata*, see Table 5 for an example language in both conditions). Noun stems from each class were randomly paired with referents for each participant. During exposure, participants heard 4 noun-referent pairings from each class, with the remaining 2 used in testing as novel nouns (see more details below).

**Table 5.** Example input structure for the two experimental conditions in Study 2.

	**No agreement**	**Agreement**	
Class 1	mibipo du	mibipo dupo	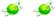
	tibipo du	tibipo dupo	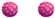
Class 2	gatafei du	gatafei dufei	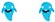
	patafei du	patafei dufei	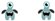

Participants were presented with images showing a single referent, described using a bare noun, and images showing a pair of the same referents, described using a modifying numeral, *du*. Unlike in Study 1, in this language the numeral always followed the noun (the reverse of English, participants’ native language). In the agreement condition, when nouns were modified by the numeral, an agreeing (and identical) suffix appeared on the numeral (e.g., *mibipo dupo*). In the no-agreement condition, the numeral had no suffix (e.g., *mibipo du*). There were 4 possible suffixes: *po*, *ka*, *zai*, *fei*. Two suffixes were randomly selected for each participant to indicate the two noun classes. The language was displayed only auditorily to participants. Words were recorded by a female native speaker of British English. See Table 6 for full language lexicon.

**Table 6.** Full lexicon of the artificial language in Study 2.

**Noun labels**		**Suffixes**	**Numeral**
mibi	gata	po	du
tibi	zata	ka	
sibi	mata	zai	
pibi	pata	fei	
dibi	kata		
kibi	nata		

#### Procedure.

The experiment was written in JavaScript using JsPsych (de Leeuw, 2015) and was conducted online. Adults participated in the study independently via the Prolific platform, completing the experiment in their own web browser, while children took part through Zoom sessions, directly interacting with an experimenter. The children viewed the experiment—which was displayed on the experimenter’s web browser and shared via screen sharing—and were instructed to direct the experimenter on which elements to click (further details are provided below). To train children for this setup, the session began with two 2AFC training trials. In each trial, two numbered images, such as a tomato and a cucumber, were displayed. The experimenter would then ask, ‘Which one is a tomato, number 1 or number 2?’ If the child could not accurately direct the experimenter to click on the correct image by its number (e.g., saying e.g., ‘this one’ or ‘the tomato’), the experimenter would repeat the question and guide the child to use the numbers. The rest of the experimental structure was identical for both children and adults. Participants were informed they would meet an alien named Tweeble and would learn its language. To motivate children, they received (virtual) medals and gold for each successful trial.

**Figure 5.**
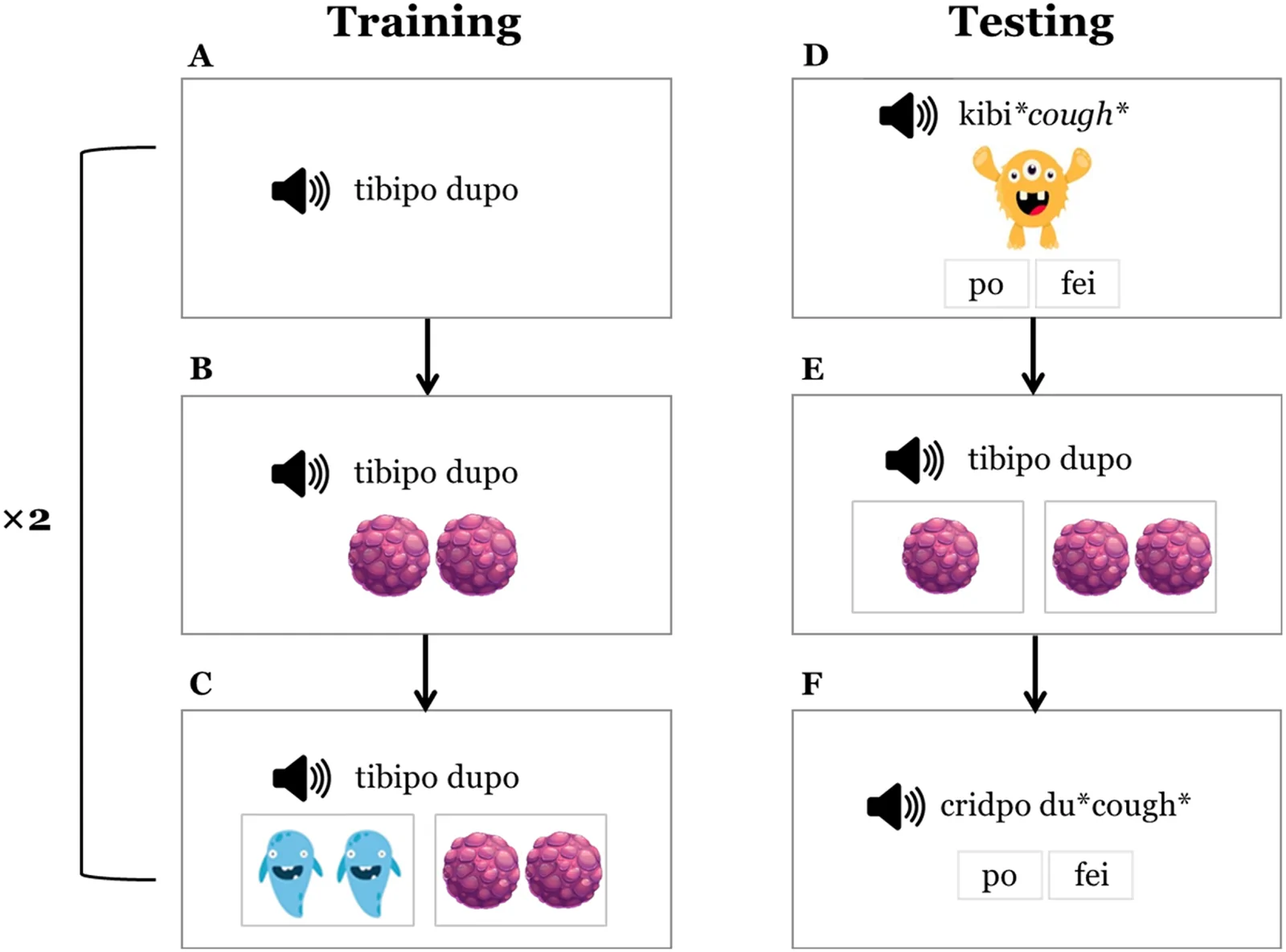
Experimental procedure for Study 2, illustrating the agreement condition. The experiment involved two training rounds, each introducing half the total nouns across three phases (A, B, C). This was followed by three testing phases: *noun classification test*, *number test*, and *agreement test* (D, E, F respectively), with the final phase (F) exclusive to the agreement condition.

Participants started with a set of three exposure training phrases. To facilitate learning, each participant went through two rounds of this training. In the first round, they went through each of the three training phases with a randomly chosen set of half of the exposure nous in each class, i.e., 2 per class. In the second round, they went through each of the three training phases with the remaining half of the nouns. As in Study 1, in the first training phase participants heard nouns without any corresponding images (see Figure 5A). Each noun appeared once in the singular and twice in the plural, for a total of 12 trials (in a given round of training). In the second training phase (Figure 5B), participants heard nouns presented along with their corresponding images. Each noun appeared once in the singular, and three times with the number, for a total of 16 trials (in a given round of training). After each trial, the participants were asked to repeat the word they just heard. The third training phase was a picture matching task (Figure 5C). In each trial, participants heard one of the exposure nouns and saw two images (or two pairs of images) on the screen. Participants had to choose which corresponded to the noun they heard. Each noun was the correct answer once in the singular and three times with the number, for a total of 16 trials (in a given round of training). The distractor images always matched the target in numerosity, but differed in class. Child participants were shown numbered images and instructed the experimenter which image to click. Adult participants saw unnumbered images, and simply clicked on their choice directly. Participants received feedback after each trial.

After completing two training rounds (i.e., going through the three training phases above twice), participants advanced to the testing phases, during which they received no feedback. The first of these was the *noun classification test*, which assessed how well participants learned to associate nouns with the correct suffixes. This test is parallel to the noun classification test in Study 1 (Figure 1E). Participants were informed that Tweeble had developed a cold that caused him to cough. In each trial, participants were shown an image of a single alien or planet, and heard a description from Tweeble (a singular noun), but his cough conveniently obscured the word ending, effectively masking the suffix. The task for participants was to identify the obscured suffix from two options: the appropriate suffix and an incorrect one associated with the other noun class. These options were presented in random order during each trial. For children, the two possible suffixes (e.g., *fei*, *po*) appeared in written form on the screen. The experimenter then presented these options out loud, combining each time the noun and the suffix following the order in which the suffixes appeared on the screen (e.g., ‘Did Tweeble mean to say *mibifei* or *mibipo*?’—see Figure 5D). The experimenter repeated the options if necessary and could select either one of the suffixes, or an empty button if the child was unsure or declined to make a choice. For adults, the two options were presented auditorily in sequence—noun and suffix together, corresponding to buttons displaying the possible suffixes. When a noun-suffix combination was spoken, the corresponding suffix on the screen was highlighted, and adults were instructed to choose the matching one. Importantly, this phase included all eight previously trained nouns *and* four novel nouns they did not hear in training. Although these novel nouns had not been introduced earlier, they followed the same classification cues as the familiar nouns: they referred to an alien or a planet, featured one of the two phonological patterns, and ended with a suffix, which, as in all trials, was obscured by Tweeble’s cough. This phase consisted of 12 trials (each of the six nouns in each class, both novel and familiar, occurred once).

The next test was the *number test*, designed to check whether participants noticed the number word and successfully learned its meaning. In each trial, participants were presented with a noun either in its singular form or with the number, alongside two images. Both images depicted the correct referent; however, one image showed a single instance of the referent, while the other showed the same referent twice. Participants were required to select the image that correctly matched the noun form presented. For children, the images were numbered, enabling them to direct the experimenter on which to click (Figure 5E). There were six such trials (three singulars, three plurals). This was the final test phase for participants in the no-agreement condition.

Participants in the agreement condition underwent an additional *agreement test*, designed to evaluate learning of the agreement patterns themselves. As such, this test targeted the agreement patterns independently of other cues: all noun stems presented were new, did not adhere to the phonological patterns of the two classes, and appeared alone, without any visual referents. The novel nouns were: *blor*, *foos*, *frag*, *crid*, *zon*, *spag*. In each trial, Tweeble presented a noun modified by a numeral; however, similar to the noun classification test, Tweeble’s cough obscured the suffix attached to the numeral (e.g., cridpo du*cough*). Participants had to identify the correct suffix from two options, which were displayed in a randomized order on the screen. This was structured similarly to the noun classification test. For children, three buttons appeared on the screen: one for each suffix option and one empty button. The options were presented out loud, with the full phrase articulated each time (e.g., ‘What did Tweeble mean to say, *cridpo dupo* or *cridpo dufei*?’). The experimenter clicked the button corresponding to the child’s answer, or the empty button if the child was unsure or declined to respond. For adults, the two options were presented auditorily one after the other, with the entire modified noun phrase played (*cridpo dupo* vs. *cridpo dufei*). Buttons displaying only the relevant suffixes corresponded to the spoken words. When a noun and suffix combination was uttered, the corresponding suffix on the screen was highlighted (Figure 5F). This phase included a total of six trials (each new noun appeared once, each suffix was used three times.

### Results

Before examining participants’ performance in the different tests, we examined their scores on the second (and final) picture-matching task during training. Adults’ average score was 93% in the agreement condition and 94% in the no-agreement condition, whereas children’s was 70% in the agreement condition and 77% in the no-agreement condition. We fit a model with accuracy as the outcome variable. Fixed effects included condition (treatment coded, with no-agreement condition as the baseline level), group (children vs. adults, treatment coded, with children as the baseline level), their interaction, and trial number (centered). Random effects included by-participant intercepts and slopes for trial number (see Table 7 for full model).

**Table 7.** Posterior means and 95% CrIs for the second picture-matching task in Study 2.

	**Estimate**	**Est’d Error**	**Lower 95% CrI**	**Upper 95% CrI**
(Intercept)	1.649	0.319	1.034	2.301
Condition = agreement	−0.512	0.433	−1.348	0.35
Group = adults	2.043	0.437	1.21	2.927
Trial number	0.031	0.022	−0.01	0.075
Condition = agreement * Group = adults	0.047	0.584	−1.12	1.197

The intercept reflects predicted accuracy for children in the no-agreement condition (83.4 pp, 95% CrI: [73.8, 90.9] pp; log-odds: *β* = 1.65, 95% CrI = [1.03, 2.30], pd = 1), indicating above-chance performance. Adults demonstrated higher accuracy than children in the no-agreement condition (14.0 pp, 95% CrI: [6.5, 23.8] pp; log-odds: *β* = 2.04, 95% CrI = [1.21, 2.93], pd = 1). This outcome is common in artificial language learning experiments (Culbertson et al., 2019; Raviv & Arnon, 2018; Tal & Arnon, 2022) and reflects the generally expected differences in performance between adults and children on the same cognitive tasks. For children, the model suggested a tendency toward lower accuracy in the agreement condition, though with considerable uncertainty (−8.1 pp, 95% CrI: [−22.3, 5.6] pp; log-odds: *β* = −0.51, 95% CrI = [−1.35, 0.35], pd = 87.8). For adults, the pattern was very similar (−8.0 pp, 95% CrI: [−24.7, 3.6] pp; log-odds: *β* = −0.46, 95% CrI = [−1.26, 0.29], pd = 88.4), with performance approaching ceiling in both conditions (93–94%). The similar magnitude of log-odds estimates between children and adults despite smaller raw percentage differences in adults likely reflects ceiling effects compressing variation at high accuracy levels. Overall, these results suggest no benefit for the agreement pattern early in learning, with a modest tendency toward lower accuracy in the agreement condition in both groups.

**Figure 6.**
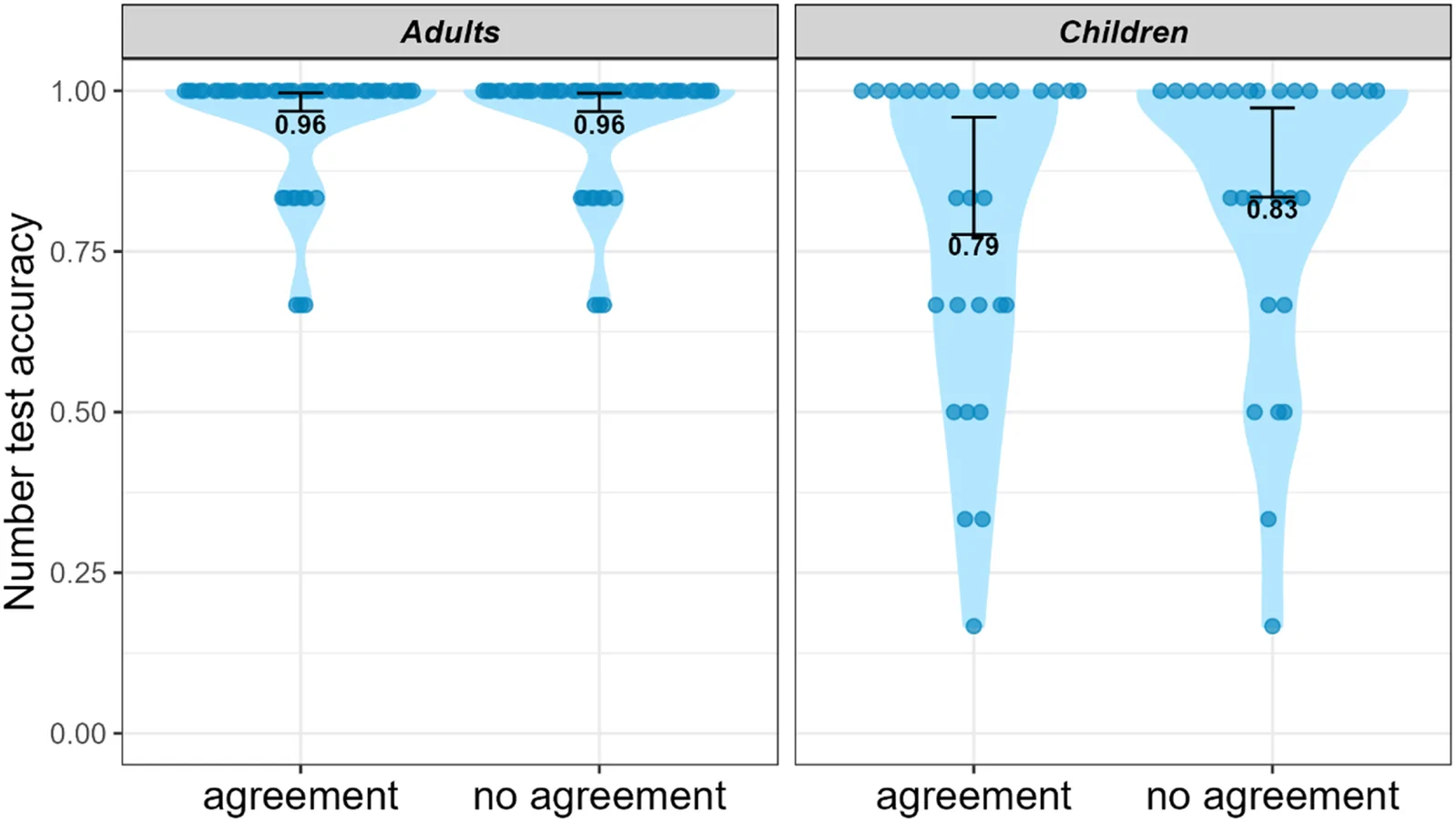
Accuracy scores in the number test by group and language condition. Error bars indicate the model’s predicted 95% credible intervals; individual points indicate by-participant means; numbers represent group means.

We then examined participants’ accuracy in the number test to ensure they noticed and understood the meaning of the numeral, and that this comprehension did not differ by condition. Figure 6 shows participants’ accuracy in choosing the correct image in the number test. We predicted accuracy in the test from condition (treatment coded, with no-agreement condition as the baseline level), group (children vs. adults, treatment coded, with children as the baseline level), their interaction, and trial number (centered). The model further included random intercepts for participants and by-participant slopes for trial number (see Table 8 for full model).

**Table 8.** Posterior means and 95% CrIs for number test in Study 2.

	**Estimate**	**Est’d Error**	**Lower 95% CrI**	**Upper 95% CrI**
(Intercept)	2.539	0.512	1.618	3.598
Condition = agreement	−0.43	0.656	−1.695	0.882
Group = adults	1.847	0.65	0.614	3.185
Trial number	0.24	0.14	−0.012	0.536
Condition = agreement * Group = adults	0.473	0.919	−1.39	2.318

The intercept reflects predicted accuracy for children in the no-agreement condition (91.9 pp, 95% CrI: [83.4, 97.3] pp; log-odds: *β* = 2.54, 95% CrI = [1.62, 3.60], pd = 1), indicating above-chance accuracy. The model also confirms that adults were more accurate than children in the no-agreement condition (6.6 pp, 95% CrI: [1.4, 15.0] pp; log-odds: *β* = 1.85, 95% CrI = [0.61, 3.19], pd = 1). With regard to the effect of condition on children, the model’s posterior places a higher probability on worse performance in the agreement condition, though with substantial uncertainty −3.6 pp, 95% CrI: [−15.3, 7.3] pp; log-odds: (*β* = −0.43, 95% CrI = [−1.7, 0.88], pd = 0.74). For adults, the posterior was essentially evenly split around zero, providing no evidence of any difference between conditions (−0.9 pp, 95% CrI: [−14.6, 7.0] pp; log-odds: *β* = 0.04, 95% CrI = [−1.19, 1.35], pd = 0.53). Taken together, on a group level, participants from both age groups and all conditions understood the meaning of the number. Thus, any influence the agreement pattern might have on noun classification is not likely to be due to variation in comprehending the numeral that carries the agreement pattern. However, as can be seen in Figure 6, five children scored below 50% in this test. Comprising only six trials, this test had no a-priori exclusion criteria predefined. All participants were thus included in the subsequent analysis of noun classification performance reported below. However, we conducted an exploratory analysis excluding the data from these children from the subsequent noun classification analyses (three from the agreement condition, two from the no-agreement condition). This analysis is reported in Appendix C. The removal of these low scores did not affect the overall pattern of results, suggesting that poor performance in this task does not significantly influence noun classification.

**Figure 7.**
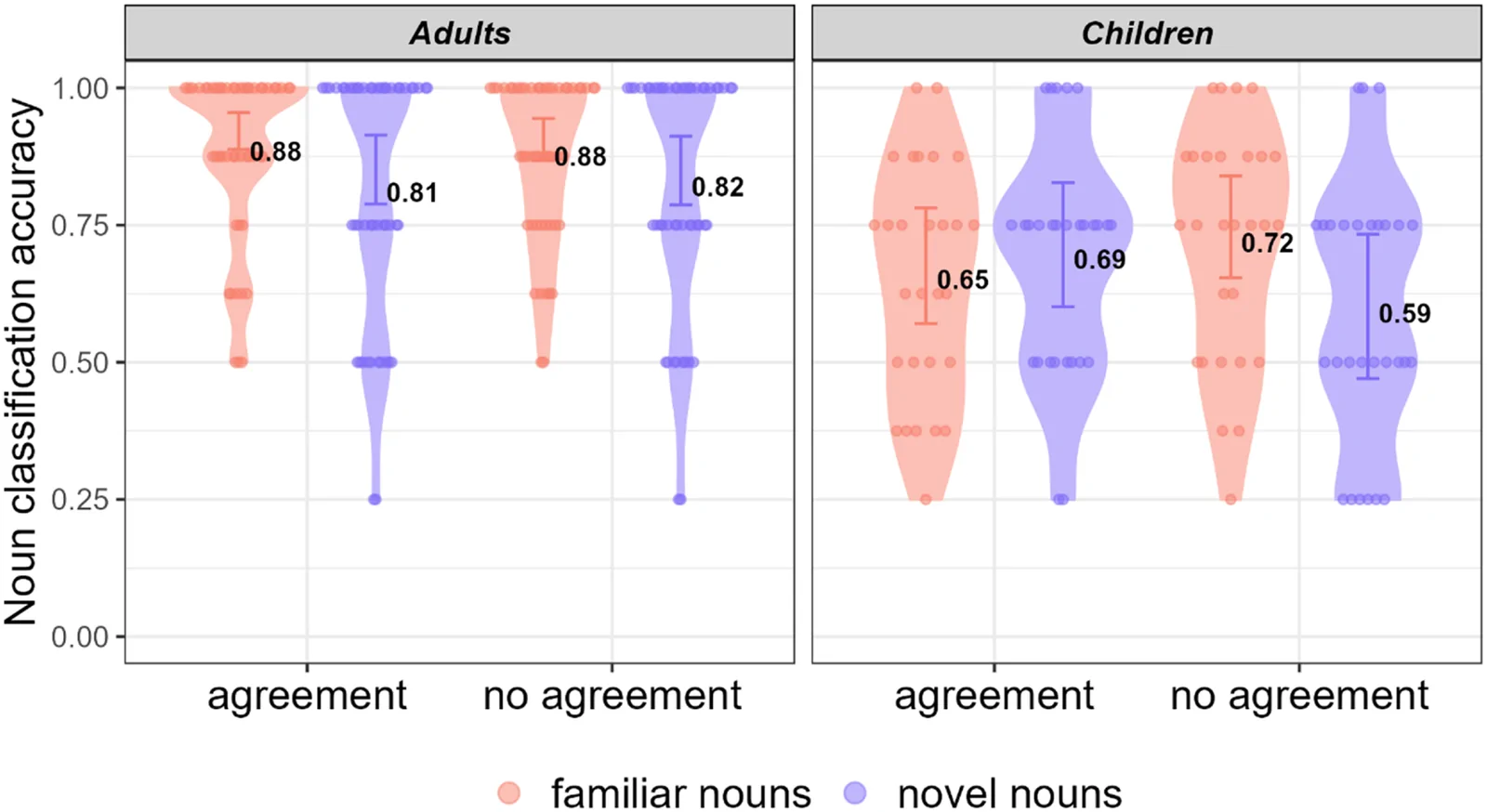
Accuracy scores in the noun classification test by group, language condition and noun type. Error bars indicate the model’s predicted 95% credible intervals; individual points indicate by-participant means; numbers represent group means.

We next turned to the key result of interest—participants’ performance in the noun classification test. Recall that in this task, participants heard a noun whose suffix was obscured, saw the corresponding referent, and were asked to identify the missing noun suffix. Figure 7 shows accuracy by group, for both familiar and novel nouns. We predicted accuracy in the test from condition (treatment coded, with no-agreement condition as the baseline level), group (children vs. adults, treatment coded, with adults as the baseline group), noun type (familiar vs. novel, treatment coded, with familiar as the baseline level), the interaction of the latter three, and trial number (centered). The model further included random intercepts for participants and by-participant slopes for noun type and trial number (see Table 9 for full model).

**Table 9.** Posterior means and 95% CrIs for noun classification test in Study 2.

	**Estimate**	**Est’d Error**	**Lower 95% CrI**	**Upper 95% CrI**
(Intercept)	2.343	0.243	1.885	2.829
Condition = agreement	0.201	0.33	−0.429	0.854
Group = children	−1.206	0.344	−1.882	−0.56
Noun type = novel	−0.523	0.27	−1.056	0.001
Trial number	−0.014	0.025	−0.062	0.036
Condition = agreement * Group = children	−0.571	0.479	−1.517	0.371
Condition = agreement * Noun type = novel	−0.208	0.38	−0.952	0.531
Group = children * Noun type = novel	−0.175	0.385	−0.938	0.557
Condition = agreement * Group = children * Noun type = novel	1.118	0.546	0.052	2.185

First, the model indicated that children achieved lower comprehension scores compared to adults (raw means: 85% vs. 66%; −12.4 pp, 95% CrI: [−20.8, −5.58] pp; log-odds: *β* = −1.21, 95% CrI = [−1.88, −0.56], pd = 1). Once again, these results align with previous studies in which adults generally outperform children in laboratory experiments. Turning to our variables of interests, using estimated marginal means to average across noun types, we found no evidence for condition effects in either adults (0.73 pp, 95% CrI: [−3.72, 5.10] pp; log-odds: *β* = −0.1, 95% CrI = [−0.7, 0.43], pd = 0.62) or children (0.34 pp, 95% CrI: [−5.39, 4.77] pp; log-odds: *β* = −0.08, 95% CrI = [−0.75, 0.54], pd = 0.59). The model also indicated uncertainty with respect to an interaction between condition and group (−0.95 pp, 95% CrI: [−11.1, 4.40] pp; log-odds: *β* = −0.57, 95% CrI = [−1.52, 0.37], pd = 0.51). However, as can be seen in Figure 7, the agreement condition appears to have impacted differently the classification of novel versus familiar nouns across the two age groups. Indeed, the model 95% CrI contains only positive values for the three-way interaction between group, condition and noun type (5.47 pp, 95% CrI: [0.29, 9.92] pp; log-odds: *β* = 1.12, 95% CrI = [0.052, 2.18], pd = 0.98), suggesting that agreement may have a stronger positive effect on novel noun classification in children than would be expected from the main effects alone.

**Figure 8.**
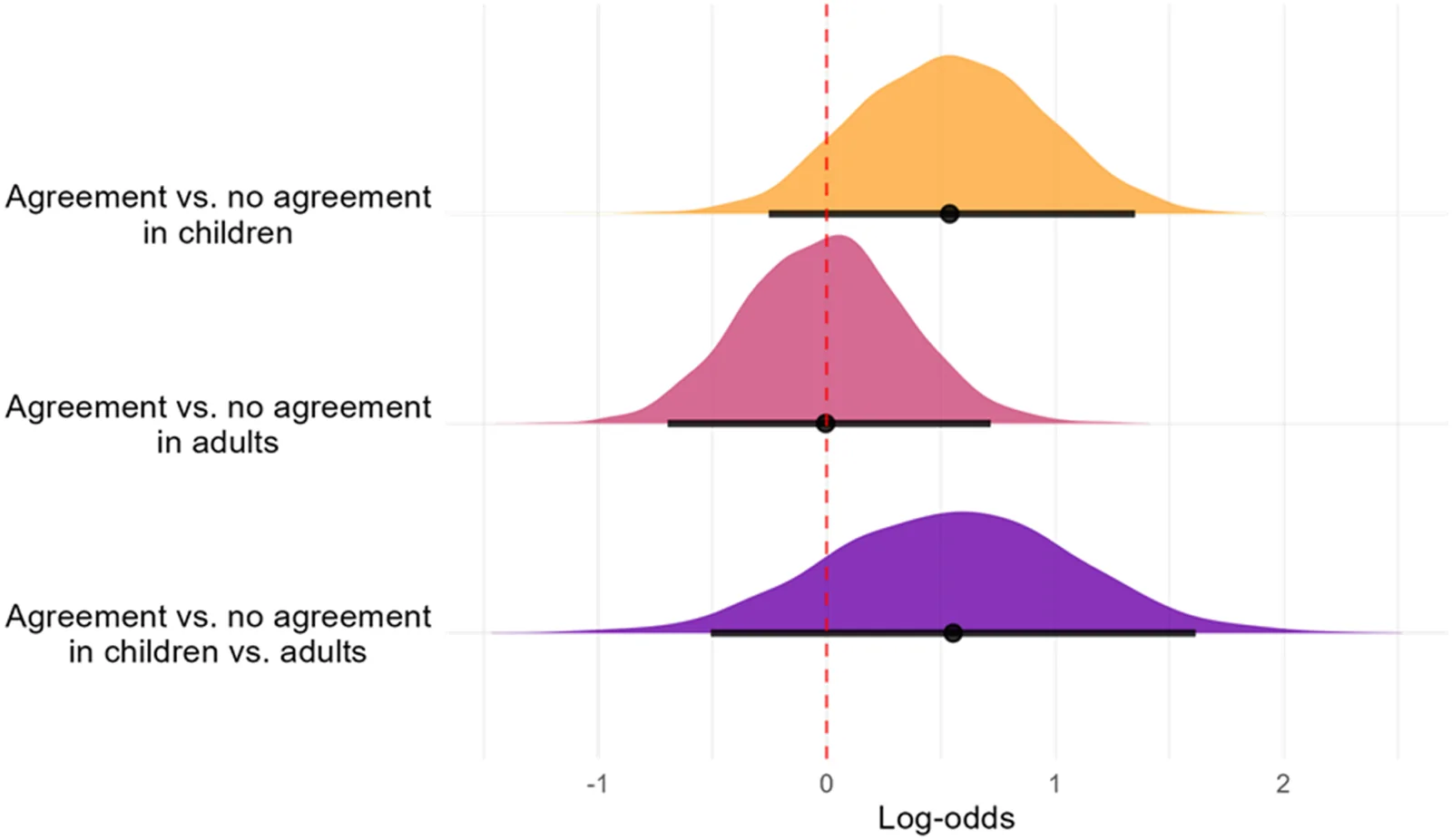
Posterior distributions of the effect of condition (agreement vs. no agreement) on accuracy in novel nouns. Top: Condition effects in novel nouns within children. Middle: Condition effects in novel nouns within adults. Bottom: Differential effect; testing whether the condition effect in novel nouns is larger in children than adults. Density curves show full posterior distributions; horizontal lines indicate 95% credible intervals. Dashed vertical line at zero represents no effect. Positive values indicate higher accuracy in the agreement condition.

To decompose this three-way interaction, we extracted posterior distributions for the effect of agreement on novel noun accuracy in each age group, along with the differential effect between the groups. Specifically, we calculated three posterior distributions: (1) the effect of condition (agreement vs. no-agreement) on accuracy for novel nouns in children, (2) the effect of condition on accuracy for novel nouns in adults, and (3) the differential effect between children and adults (i.e., the difference between (1) and (2)). These posterior distributions are visualised in Figure 8.

For children, agreement was associated with higher novel noun classification accuracy (11.7 pp, 95% CrI: [−5.49, 28.69] pp; log-odds: *β* = 0.54, 95% CrI = [−0.25, 1.35], pd = 90.2). The credible interval is nonetheless consistent with effects ranging from negligible to quite large, reflecting uncertainty about the magnitude of the effect. For adults, the effect of agreement on novel noun accuracy is negligible (−0.08 pp, 95% CrI: [−8.48, 8.7] pp; log-odds: *β* = −0.01, 95% CrI = [−0.70, 0.72] pd = 0.50), with the posterior essentially evenly split around zero, providing no evidence of any systematic effect in either direction. Finally, the differential effect between groups—the degree to which the agreement benefit is greater in children than adults—also favours a positive direction (11.8 pp, 95% CrI: [−7.73, 31.0] pp; log-odds: *β* = 0.55, 95% CrI = [−0.51, 1.62], pd = 0.88). Taken together, these results indicate a probable facilitatory effect of agreement on novel noun classification in children, alongside no discernible tendency in adults.

**Figure 9.**
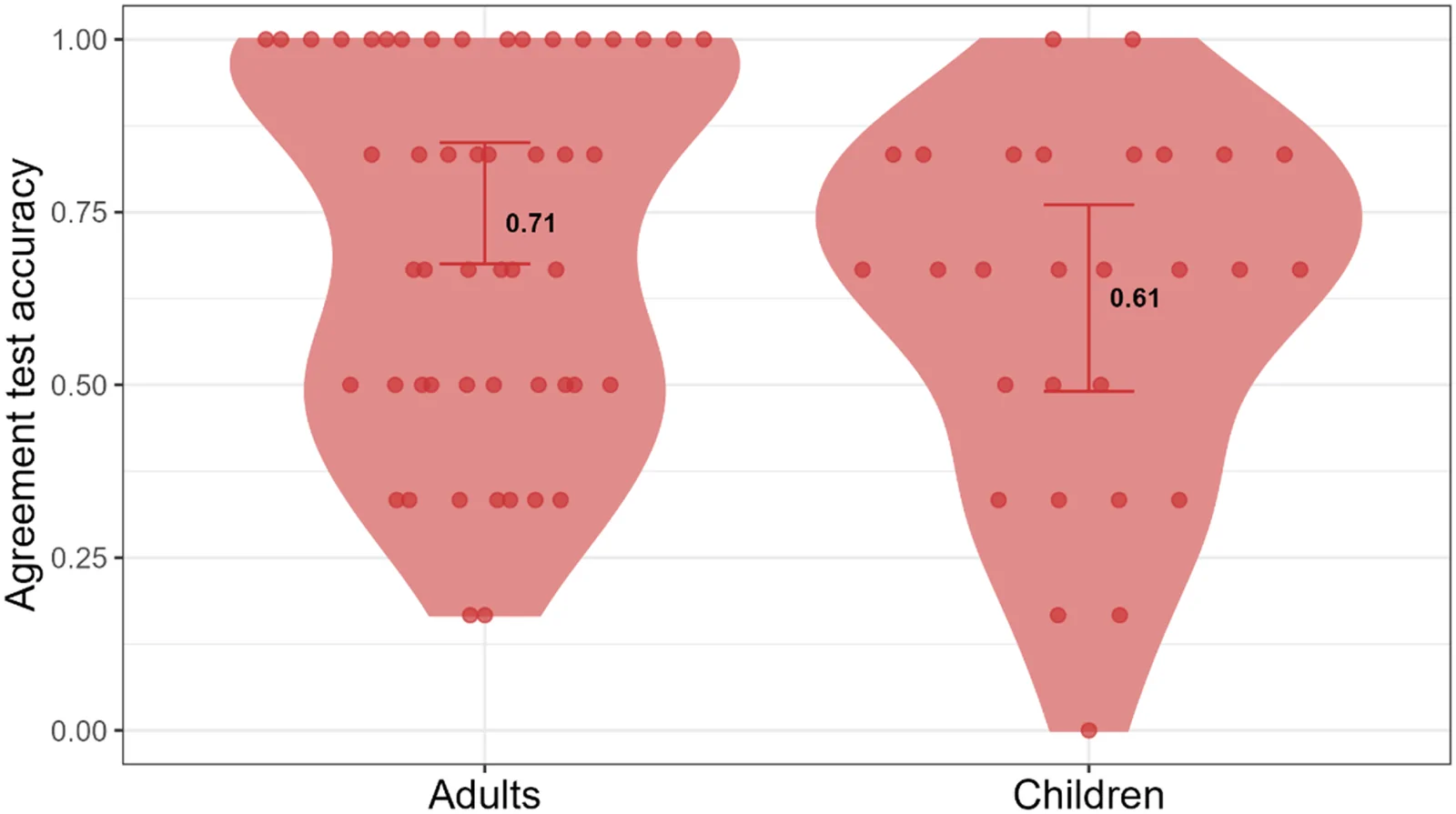
Accuracy scores in the agreement test by group. Error bars indicate the model’s predicted 95% credible intervals; individual points indicate by-participant means; numbers represent group means.

In our final analysis, we examined participants’ performance in the agreement test, which assessed how well participants in the agreement condition learned the agreement patterns themselves. The accuracy of participants in this test is depicted in Figure 9. We predicted accuracy using group (treatment coded, with children as the baseline level) and trial number (centered) as predictors. The model also included by-participant slopes for trial number and by-participant intercepts (see Table 10 for the full model). The intercept reflects predicted accuracy for children, estimated at 63.4 pp (95% CrI: [49.1, 76.1] pp; log-odds: *β* = 0.56, 95% CrI = [−0.04, 1.16], pd = 96.5). With 96.5% of the posterior above zero, this indicates above-chance performance, though with some uncertainty. Adults performed above chance, and more conclusively so (76.5 pp, 95% CrI: [67.5, 85.1] pp; log-odds: *β* = 1.2, 95% CrI = [0.73, 1.74], pd = 1). Notably, however, both age groups were far from excelling this test. We will discuss this in the next section.

**Table 10.** Posterior means and 95% CrIs for agreement test in Study 2.

	**Estimate**	**Est’d Error**	**Lower 95% CrI**	**Upper 95% CrI**
(Intercept)	0.56	0.302	−0.037	1.156
Group = adults	0.64	0.377	−0.072	1.408
Trial number	−0.049	0.081	−0.203	0.112

### Discussion

Study 2 explored the influence of agreement marking on learning a novel noun classification system in children compared to adults. The results indicate that, overall, children did not benefit from agreement marking more than adults. However, we found that children and adults likely respond differently to agreement marking. Specifically, children may classify novel nouns more accurately when agreement is present compared to when it is not. By contrast, adults showed no such effect; they consistently performed better with familiar nouns, regardless of the presence of agreement marking. We discuss how this possible difference might be interpreted in the General Discussion.

The results of the number test showed that overall, both children and adults understood the meaning of the number word. However, a few children received low scores on this test. Since the pattern of results did not change when these children were excluded from analysis (see Appendix C), this suggests that, at least for children, noun classification may not depend on comprehension of the dual. This interpretation is in line with previous studies showing that children tend to rely more on phonological cues and less on semantic cues in noun classification (Culbertson et al., 2019).

Finally, while adults performed above chance in correctly matching the suffix on the numeral to the suffix on the noun during the agreement test, children’s performance was uncertain, and potentially not better than chance. Notably, neither group excelled in this test. The results are particularly revealing when compared to the results from Study 1, where participants in the alliterative-agreement condition—the same type used in the current study—achieved an average score of 95%. In contrast, adults in Study 2 scored only 71%. We believe this discrepancy suggests that the agreement test in Study 2, despite being designed to be child-friendly, was in fact quite challenging. Recall that in this test, participants heard a phrase with the suffix on the numeral obscured by a cough (e.g., cridpo du*cough*). They were then presented with two auditory options for the full phrase sequentially (*cridpo dupo*, *cridpo dufei*). Therefore, the test required participants to retain both options in memory, a task that might be more taxing and less intuitive, especially for children, compared to completing a phrase with a missing suffix as in Study 1.

## GENERAL DISCUSSION

The widespread presence of agreement patterns in the world languages, despite the complexity they add (Corbett, 2006; Leufkens, 2020, 2023), suggests they might offer some cognitive or functional advantages. Research has indeed identified several potential benefits of agreement for language users. For example, agreement can aid proficient language users in predicting upcoming information (Dye et al., 2017). Here, we investigated the possibility that agreement can be beneficial even before a comprehensive knowledge of the language is developed. That is, we explored whether agreement, as a manifestation of linguistic redundancy, can be beneficial for language learning.

The Linguistic Niche Hypothesis (Lupyan & Dale, 2010) proposes that child learners in particular should benefit from redundancy, while adult learners might not. This hypothesis has received support from a recent artificial language learning study, showing that child learners are more successful at learning thematic role assignment in a language in which both word order *and* a redundant morpheme provide cues (Tal & Arnon, 2022). Whether adults also benefitted from a redundant morpheme was however not clear.

Here, we investigated whether agreement marking can confer an advantage for learners acquiring a novel noun classification system. In Study 1, we focused on adult learners. We compared how well participants learned across three different versions of the artificial language: one without any agreement, one with alliterative agreement (where the agreeing forms are identical), and one non-alliterative agreement (where the agreeing forms are not identical). We also compared between nouns with varying degrees of evidence for noun classification; some nouns had a semantic cue, while other did not. Our findings provided little evidence for a learning advantage associated with agreement marking, regardless of the presence of semantic cues. However, in line with predictions that alliterative agreement would be less cognitively demanding, we found that the agreement pattern itself was easier to learn when it was alliterative rather than non-alliterative.

To examine differences between child and adult learners, Study 2 used a simplified artificial language, either without agreement or with alliterative agreement. Unlike in Study 1, all nouns in this study had a semantic cue, making the cues for noun classification deterministic. However, similar to Study 1, the evidence available for noun classification varied: some nouns were familiar from the training phase, while others were novel. The results revealed that, as in Study 1, adults showed no difference between the conditions. Contrary to our predictions, there was also no significant difference observed in children. Nonetheless, we found that children and adults were potentially impacted differently by the language condition manipulation: agreement marking may have specifically facilitated the classification of novel nouns in children, whereas it had no discernible effect on adults. Importantly, our statistical analysis indicated that while most of the posterior probability pointed to such a difference between children and adults, there was substantial uncertainty. Thus, we cannot conclusively determine whether agreement marking aids children’s classification of novel nouns in this task. It should be noted that this study provided children with only brief exposure to the language, which differs considerably from real-life language acquisition (see discussions in Culbertson & Schuler, 2019; Tal et al., 2025). It is possible that increasing exposure would reveal stronger effects. However, the fact that probable differences were found between children and adults after such a short duration lends support to the idea that children and adults might learn differently from these cues.

While further research is needed to determine whether this difference holds robustly, it is nevertheless worth considering why the effect of agreement might specifically benefit classification of novel nouns in children. Although the novel nouns in Study 2 followed the same obligatory cues for noun classification as familiar nouns, they posed greater challenges because they were not presented during training, preventing rote memorization. For these nouns, the additional agreeing suffix on the numeral could serve as a helpful cue for children. However, our findings suggest that such an additional cue does not benefit adults. For adults, redundant morphemes were not helpful when most needed: neither in cases where nouns lacked a semantic cue (Study 1), nor when the nouns were novel (Study 2). Indeed, adults generally struggle with acquiring morphology (Clahsen et al., 2010; DeKeyser, 2005; Ellis, 2022), and tend to rely on other cues when these are available (Kenanidis et al., 2023; Pankratz et al., 2024). This raises the possibility that children and adults might not only differ in their ability to acquire morphology, but also in how they learn *from* morphology. Such potential differences between children and adults prompt several important questions. First, what underlies these differences? A prominent theory suggests that morphological cues are less salient to adult second-language learners (Ellis, 2022), however, it remains unclear why children in a similar second language learning context appear to behave differently. Secondly, might adults benefit from redundant cues that are not morphological? Evidence involving multi-modal cues supports this possibility (Murgiano et al., 2021), though it is unclear whether redundant non-morphological linguistic cues can hold similar benefit. We are currently investigating this possibility in ongoing work. Finally, if young children, unlike adults, benefit from redundant morphology, this advantage might be present from an early age and guide the initial stages of language acquisition. We are currently investigating whether redundant morphology also benefits infants (see Gerken et al., 2005 for some supporting evidence). Taken together, this study contributes to the growing body of literature indicating that children and adults might rely on different types and scopes of cues during language acquisition (Arnon, 2021; Berger & Batterink, 2024; Culbertson et al., 2019; Hudson Kam & Newport, 2009).

The present findings also have implications for the literature on language complexity. The Linguistic Niche Hypothesis (Lupyan & Dale, 2010) posits that child and adult learners learn differently from redundant morphology, with children benefitting from it but not adults. Lupyan and Dale (2010) propose this difference as a possible explanation for why languages that are commonly used by adults tend to lose morphological complexity over time (Bentz & Winter, 2013; Dale & Lupyan, 2012; Lupyan & Dale, 2010; Trudgill, 2009, 2011). Previous work identified a benefit of redundant morphology for children (Tal & Arnon, 2022). The results of Study 2, though somewhat uncertain, suggest that children and adults may indeed learn differently from redundant morphology. If children benefit from redundancy while adults do not, this could explain why languages maintain these forms despite the added complexity.

Finally, if redundant morphology could be beneficial for child learners, is it also more prevalent in speech *directed* to them? Growing evidence indicates that speech directed to younger children tends to be more redundant across several domains: referential choice (Tal et al., 2023), lexical repetition (Nencheva et al., 2024; Soderstrom, 2007), frequent frames (Cameron-Faulkner et al., 2003; Stoll et al., 2009) and overall input (Tal et al., 2024). If speakers introduce more redundancy in their speech when talking to young children, this should also manifest in increased morphological redundancy. Studies examining cues to thematic assignment support this hypothesis, showing that speech directed to children often includes overlapping cues to clarify ‘who is doing what to whom’ (Ibbotson & Tomasello, 2009). However, more research is needed on languages with optional morphological redundancy. In such languages, child-directed speech is predicted to feature more redundant morphemes compared to adult-directed speech.

## FUNDING INFORMATION

This research received funding from the European Research Council (ERC) under the European Union’s Horizon 2020 Research and Innovation Programme (grant agreement 681942, awarded to KS, and grant agreement 757643, awarded to JC), and by the Royal Society of Edinburgh (RSE) Small Research Grant (award number 4937, awarded to ST).

## AUTHOR CONTRIBUTIONS

S.T.: Formal analysis; Methodology; Software; Visualization; Writing – original draft. K.S.: Conceptualization; Methodology; Writing – review & editing. J.C.: Conceptualization; Methodology; Writing – review & editing.

## DATA AVAILABILITY STATEMENT

The data for this paper and the analysis code used to generate all reported results are available here: https://osf.io/y695j.

## Notes

^1^ See Tal and Arnon (2019) for an analysis of the results from children alone.^2^ The language stimuli was taken from a previous noun class learning experiment by Culbertson et al. (2017).^3^ We also conducted a variant of the experiment featuring a test involving a different type of noun classification, where participants were presented with single referents next to their corresponding singular noun with a missing suffix (e.g., *sput_*). Participants had to choose between two suffix options: the correct suffix and a suffix from another class (e.g., *pa* vs. *ki*). This version was also run on 50 adults in each condition, yielding a pattern of results similar to those reported here.

## Figures and Tables

**Figure C1.**
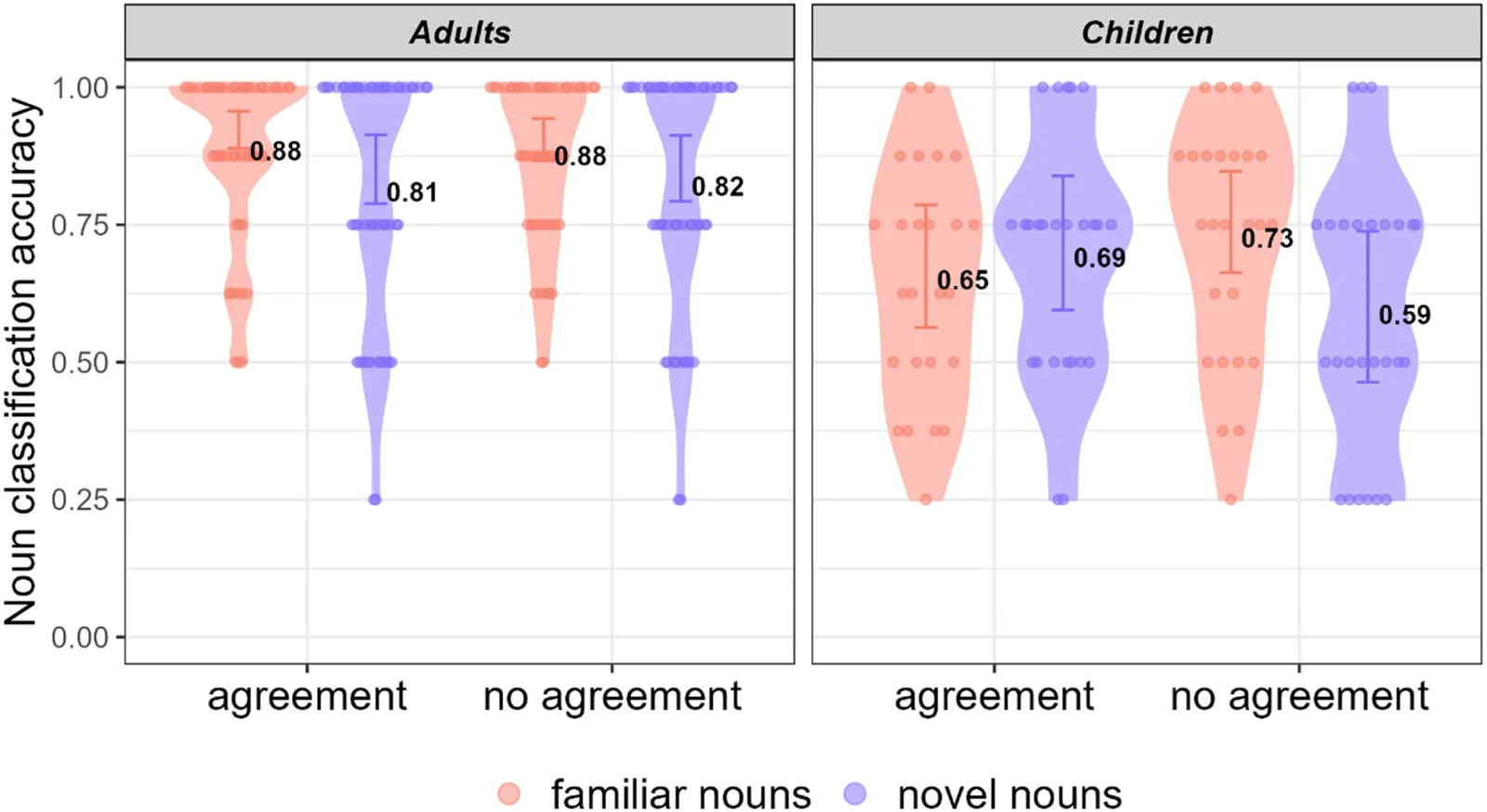
Accuracy scores in the noun classification test by group, language condition and noun type, excluding children that received low scores in the number test. Error bars indicate the model’s predicted 95% credible intervals; individual points indicate by-participant means; numbers represent group means.

**Figure C2.**
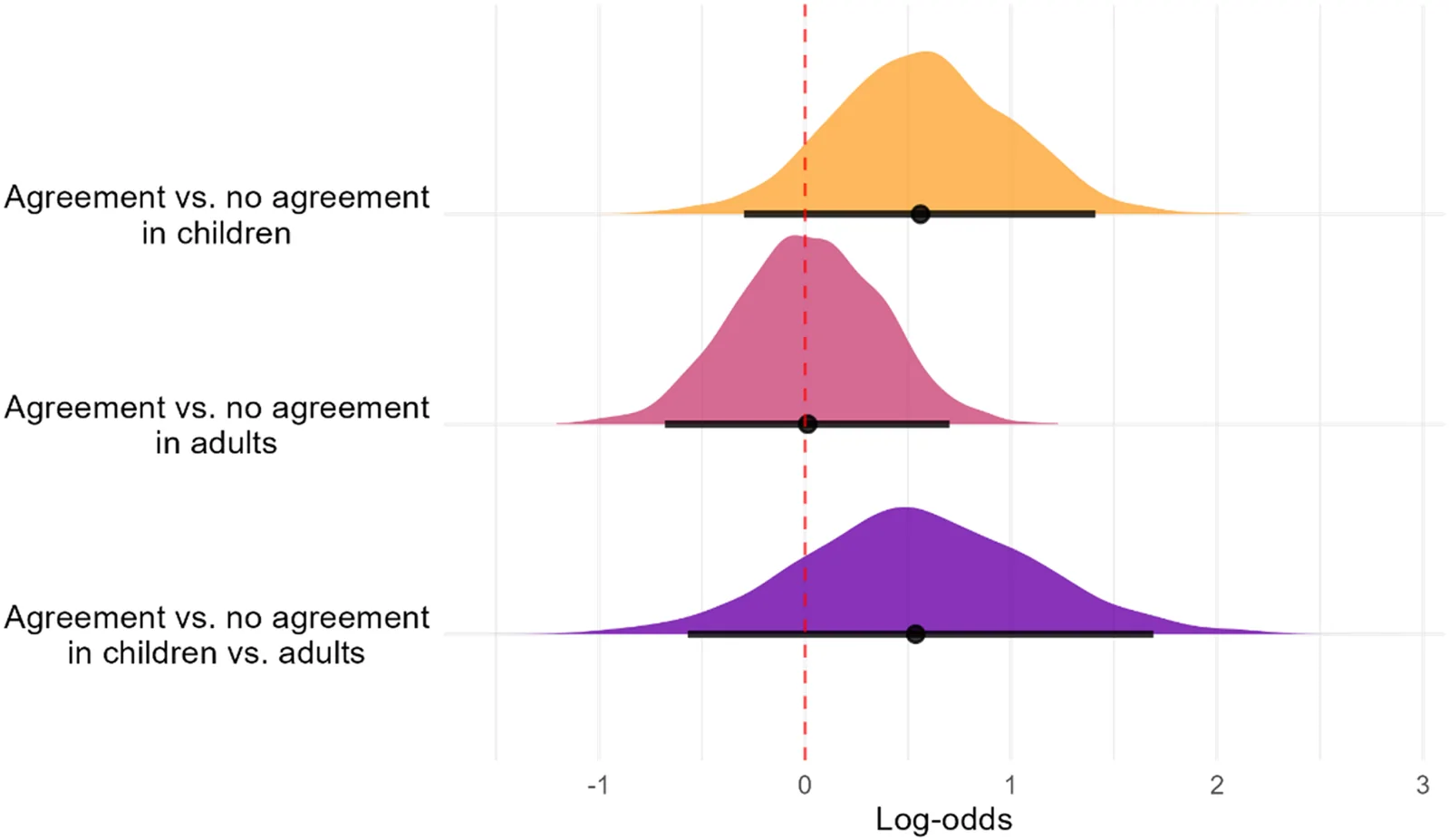
Posterior distributions of the effect of condition (agreement vs. no agreement) on accuracy in novel nouns, excluding children that received low scores in the number test. Top: Condition effects in novel nouns within children. Middle: Condition effects in novel nouns within adults. Bottom: Differential effect; testing whether the condition effect in novel nouns is larger in children than adults. Density curves show full posterior distributions; horizontal lines indicate 95% credible intervals. Dashed vertical line at zero represents no effect. Positive values indicate higher accuracy in the agreement condition.

## APPENDIX A

**Table A1.** Referents used in Study 1.

Animals		Plants		Objects
cat	shark	bamboo	cherry-blossom	guitar
fox	frog	bush	rose	hat
dog	pigeon	cactus	spikelet	hammer
elephant	squirrel	cane	succulent	lamp
cow	panda	daisy	sunflower	spoon
monkey	peacock	dandelion	tulip	umbrella
horse	raccoon	orchid	waterlily	camera
kangaroo	whale	palm	willow	piano
giraffe	butterfly	pine	dahlia	pan

## APPENDIX B

**Table B1.** All possible lexical items in the artificial language in Study 1. Of these, a random set of 18 nouns for exposure, 24 nouns for testing, and one numeral were chosen for each participant. The number of suffixes was determined based on condition, but in all cases, suffixes were randomly mapped to classes.

Noun labels			Suffixes	Numerals
forch	lour	crail	ki	gleaf
nuf	cust	trist	pa	glurn
foos	yort	crin	mu	
zear	blor	sput	ze	
mer	veen	stoon		
jeal	zast	skern		
deet	crid	pej		
vaid	tars	noaf		
roaf	parn	von		
sleaf	lij	zon		
teef	clad	vid		
clut	voast	spag		
truf	toj	zoat		
cloon	pors	frag		

## APPENDIX C

The results of the number test revealed that five children scored below 50%: three in the agreement condition and two in the no-agreement condition. In this section, we apply the same models and analyses used for the noun classification test described in the main text for Study 2 excluding data from these five participants.

Figure C1 presents accuracy by group, condition and noun type. Table C1 summarises the posterior distributions of the effects estimated by the model. The estimated effects remain qualitatively the same: we find no evidence for an overall benefit for the agreement condition or interaction between agreement and group, but we find evidence for a positive three-way interaction effect between agreement, group and noun type. To investigate this interaction, we extracted the same posterior distributions as in the original analysis, and found the same pattern of results. The posterior distributions are visualised in Figure C2.

**Table C1.** Posterior means and 95% CrIs for noun classification test in Study 2 excluding children that received low scores in the number test.

	**Estimate**	**Est’d Error**	**Lower 95% CrI**	**Upper 95% CrI**
(Intercept)	2.316	0.239	1.873	2.813
Condition = agreement	0.222	0.331	−0.425	0.893
Group = children	−1.128	0.355	−1.84	−0.454
Noun type = novel	−0.509	0.269	−1.035	0.034
Trial number	−0.011	0.025	−0.058	0.042
Condition = agreement * Group = children	−0.637	0.504	−1.642	0.369
Condition = agreement * Noun type = novel	−0.209	0.38	−0.968	0.53
Group = children * Noun type = novel	−0.24	0.394	−1.01	0.541
Condition = agreement * Group = children * Noun type = novel	1.187	0.562	0.102	2.292
